# Capacity development among academic trainees in community-based primary health care research: The Aging, Community and Health Research Unit Experience

**DOI:** 10.1017/S1463423619000732

**Published:** 2019-10-30

**Authors:** Rebecca Ganann, Shelley Peacock, Anna Garnett, Melissa Northwood, Ashley Hyde, Sue Bookey-Bassett, Laurie Kennedy, Maureen Markle-Reid, Jenny Ploeg, Ruta Valaitis

**Affiliations:** 1 Assistant Professor, School of Nursing, McMaster University, Hamilton, Ontario, Canada; 2 Assistant Professor, College of Nursing, University of Saskatchewan, Saskatoon, Saskatchewan, Canada; 3 PhD student, School of Nursing, McMaster University, Hamilton, Ontario, Canada; 4 PhD candidate, Faculty of Nursing, University of Alberta, Edmonton, Alberta, Canada; 5 Assistant Professor, Daphne Cockwell School of Nursing, Ryerson University, Toronto, Ontario, Canada; 6 School of Nursing, Aging, Community and Health Research Unit Administrator, McMaster University, Hamilton, Ontario, Canada; 7 Professor, School of Nursing, McMaster University, Hamilton, Ontario, Canada

**Keywords:** capacity development, interdisciplinary research, training and mentorship

## Abstract

Health care system capacity and sustainability to address the needs of an aging population are a challenge worldwide. An aging population has brought attention to the limitations associated with existing health systems, specifically the heavy emphasis on costly acute care and insufficient investments in comprehensive primary health care (PHC). Health system reform demands capacity building of academic trainees in PHC research to meet this challenge. The Aging, Community and Health Research Unit at McMaster University has purposefully employed a capacity building model for interdisciplinary trainee development. This paper will describe the processes and outcomes of the model, outlining how the provision of funding, mentorship, and a unique learning environment enables capacity building in networking, collaboration, leadership development, and knowledge mobilization among its trainees. The reciprocal advancement of the research unit through the knowledge and productivity of trainees will also be detailed.

## Background

The recent Astana Declaration states that strengthening primary health care (PHC) requires research, the application of scientific knowledge, and capacity building (World Health Organization, 2018). Unfortunately, strategies to build capacity in PHC research trainees are lacking in the literature (Stewart *et al.*, 2010). An aging population has brought attention to the limitations associated with existing health systems, specifically the heavy emphasis on costly acute care and insufficient investments in comprehensive PHC. Unsustainable funding models, together with public preferences to *age-in-place*, drive the need for health system innovations to support care delivery models to better address the needs of older adults. Health system reform is required and a concomitant need to build capacity in PHC research to support system transformation. McMaster University’s Aging, Community and Health Research Unit (ACHRU) has strategically engaged trainees, patient and public research partners, community-based service providers, and policymakers in its research program with a goal of building PHC and patient-oriented research capacity.

## Aim

The aim of this paper is to describe ACHRU’s capacity building strategy for early career interdisciplinary PHC trainees.

## Context

The goal of the ACHRU research program is to promote optimal aging at home for older adults with multiple chronic conditions (≥2; MCC). Established in 2013, ACHRU seeks to address gaps in PHC and integrated person-centered care for older adults living with multimorbidity (Markle-Reid *et al.*, 2018). The research program codesigns, implements, and evaluates new community-based interventions to improve the quality of life and care of this population. The research program was conceptualized and aligned with the Knowledge-to-Action Framework (Graham *et al.*, 2006) and the Complexity Model (Grembowski *et al.*, 2014); it is guided by a pragmatic paradigm and principles of holistic person and family-centered care, collective impact, patient and public engagement, codesigning, integrated knowledge translation, mutual respect, inclusive mechanisms, and valuing all contributions. Research program approaches include user-centered design, intervention evaluation in alignment with the Quadruple Aim Framework (Bodenheimer and Sinsky, 2014), implementation science, patient-oriented research, and capacity development. The program was implemented in Ontario and Alberta, engaging trainees across multiple universities.

In 2007, a study evaluating Canada’s PHC capacity identified a need for coordinated research strategy to inform health system redesign, targeted PHC funding, and strategic capacity development for PHC clinicians and researchers (Russell *et al.*, 2007). The Canadian Institutes of Health Research (CIHR) Signature Initiative in Community-Based Primary Healthcare (http://www.cihr-irsc.gc.ca/e/43626.html) was launched in response to this report (Wong *et al*., 2018), funding 12 cross-jurisdictional and interdisciplinary teams across Canada. These teams would collaborate in studying innovative approaches that could improve the delivery of appropriate and high-quality community-based person-centred PHC to all Canadians; the ACHRU was one of the teams funded by the initiative, with additional support from the Ontario Ministry of Health and Long-Term Care, Health System Research Fund. ACHRU launched with three foundational descriptive studies seeking to better understand the issues facing older adults living with MCC and their caregivers. These studies informed the design of three subsequent intervention studies; ACHRU has since expanded to include 13 core studies and multiple leveraged studies (Markle-Reid *et al*., 2018). This rich and extensive program of research brought together multiple stakeholders (ie, policymakers, patients, caregivers, providers, health care researchers, postdoctoral fellows, graduate and undergraduate students), providing many opportunities for building patient-oriented research capacity among PHC trainees.

### Capacity development approach

**Figure 1.**
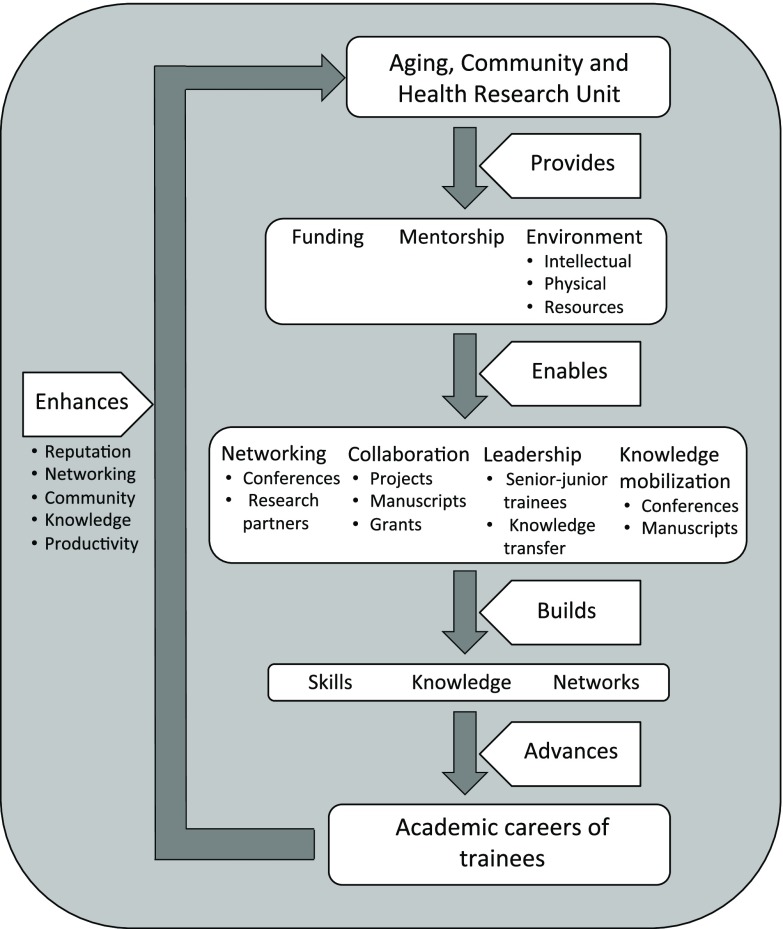
Aging, Community and Health Research Unit capacity building model of trainee development

To date, ACHRU has engaged over 60 trainees representing diverse disciplines (eg, health sciences, economics, business, engineering) from 5 Canadian universities across 4 provinces. ACHRU was purposeful in designing a learning environment to foster capacity building among its trainees. The model in Figure 1 illustrates building blocks for capacity building and the resultant key areas of growth and development. A critical early activity was to create the ACHRU Capacity Building Committee to drive the trainee-led initiatives and engage new students. This committee was supported by ACHRU’s scientific directors and program administrator and began with a faculty member, postdoctoral fellow, and a PhD student. Committee membership changed with evolving individual responsibilities, which offered opportunities to other trainees. The faculty member remained to provide continuity and mentor the committee. Capacity Building Committee activities were entirely trainee-driven and directly supported the core objectives of the research program.

A key strategy to sustain a trainee-driven environment was developing and implementing a seminar series that provided opportunities for trainees to present and learn from the team’s expertise. Capacity building activities were promoted through regular email communication. Scheduling of these activities was varied to accommodate multiple time zones and avoid conflicts with other learning opportunities. The ACHRU trainees had varying skill level foundations in PHC research. The capacity building strategy provided opportunities to expand knowledge and skill while fostering collegiality and commitment to research and trainee success. Additionally, peer mentorship frequently complemented formal mentorship structures (eg, fellows mentoring graduate students).

Every effort was made to develop capacity across jurisdictions (eg, using *WebEx* afforded distance participation in seminars and workshops); however, cross-provincial capacity building was hindered by factors such as the ability to access appropriate technology/IT personnel, as well as reduced opportunities for informal mentorship activities. Nevertheless, feedback from out-of-province trainees revealed that they found benefit when attending the capacity building seminars, which exposed them to different academic environments and other experts in PHC.

Capacity building strategies evolved over time to address trainee-identified needs and refine approaches based on feedback. ACHRU’s trainees experienced capacity development opportunities primarily within ACHRU’s research program but many extended their development through participation and leadership roles within the Signature Initiative’s strategy to build capacity across teams (http://www.cihr-irsc.gc.ca/e/50460.html). This broader strategy also included Capacity Building Webinars for trainees where several ACHRU trainees presented their own research and others presented on behalf of the ACHRU research program. Leadership roles in the Cross-Teams Working Group (RG, SBB, AG, MN) fostered networking with trainees and academic researchers across the 12 teams, increased their professional profiles, and provided opportunities to coauthor presentations and publications.

ACHRU trainees gained rich opportunities resulting in a pan-Canadian network of early career PHC researchers. Many students began without specific interest in PHC and aging research but left continuing to pursue PHC research with older adults. ACHRU has made significant investments in supporting trainees as they prepare for positions as academic and health system leaders as can be seen in ACHRU’s capacity building video (tinyurl.com/ACHRU-cb-video) and select quotes from trainees’ letters of support for faculty mentorship and teaching awards:

Working as a trainee has enabled me to attend international conferences, participate in roundtables with stakeholders, researchers and study participants, and engage in multiple aspects of research such as data collection, analysis, and manuscript preparation.

[ACHRU] fostered an environment of inclusion whereby undergraduate, graduate and post-graduate students are encouraged to participate in all aspects of the research unit. This unique approach to mentorship helps develop future academics who are extremely capable and have developed the necessary skills and confidence required to develop, implement and realize the full potential of a research idea.

Two capacity development frameworks have been used to map ACHRU’s trainee capacity development strategy and outcomes: (1) CIHR Strategy for Patient-Oriented Research Capacity Development Framework (Canadian Institutes of Health Research, 2015); and (2) Bornstein and colleagues’ (2018) set of core competencies for health services and policy research for graduate-level training programs (see Table 1).

**Table 1.** Capacities and strategic mechanisms

Capacities	Strategic mechanisms (select examples)
Health services and policy research competencies (Bornstein *et al*., 2018)	
Understanding health systems and the policy-making process	Engaging trainees in research program events fostered connections with Canadian and international PHC researchers, providers and policymakers Funding and administrative support for trainee-led capacity building events (eg, guest speaker from CIHR re: grant/scholarship landscape)
Integrated Knowledge translation (KT) activities tailored to the specific needs of PHC clinicians and policymakers	Providing opportunities for co-/lead authorship of peer-reviewed publications, research briefs, government reports, and presentations Providing KT workshop; engagement in study-specific KT planning Implementing integrated KT approach to foster learning across knowledge-user groups; trainee-led study to evaluate this approach
Networking	Developing cross-provincial trainee-led and administratively supported capacity building initiative Funding trainee participation in national/international conferences, face-to-face cross-provincial KT events with practice and local/provincial policy decision-makers, and researchers Hosting informal networking events
Negotiation and dialog	Involving postdoctoral fellows in strategic communication with policymakers and partnership negotiation meetings with research sites
Project management	Engaging trainees in planning and implementing stakeholder, scientific, and advisory committee meetings, KT plans
Interdisciplinary collaborations among patients, researchers, health practitioners, and policymakers	Partnering diverse interdisciplinary researchers with trainees and policymakers providing many opportunities for knowledge exchange Direct engagement between trainees and patient and public research partners
Change management and implementation	Engaging trainees in research team meetings offered valuable insights into real-world implementation, strategic and collaborative problem-solving, and dynamic practice/policy contexts
Leadership, mentorship and collaboration	Committing to developing leadership and mentorship Designating ACHRU administrator support for trainee engagement (eg, orientating new trainees, maintaining email distribution list, profiling trainees’ success)
Analysis and evaluation of health-related policies and programs	Developing knowledge and skills from experiential opportunities in research planning, implementation and evaluation, and translation
Patient-oriented research competencies **(**Canadian Institutes of Health Research, 2015 **)**	
Ensuring capacity for meaningful patient engagement	Hosting foundational Patient and Public Engagement in Research workshop Supporting participation in Masterclass in Patient-Oriented Research Proving experiential opportunities to work directly with Patient and Public Research Partners
Mobilizing existing expertise	Facilitating access to PHC networks and complementary capacity development programs Leveraging investigator, trainee, and research staff expertise through workshops and seminars
Supporting careers (incl. training and mentorship opportunities)	Planning for capacity building in grants, aims, and Terms of Reference Assessing learning needs; strategies tailored to meet trainee needs Providing interactive workshops and seminars on topics such as, innovative research methodologies, research skill development, writing for publication, patient engagement, information and resource sharing, strategic career development, and networking Significantly investing in graduate student and postdoctoral salary support; research assistantships Instrumentally supporting external research and scholarship applications
Building capacity to apply research to real-world problems	Embedding trainees throughout governance structure and study teams Creating opportunities to engage with patient, caregiver, practice, and policy research partners to better understand and align research with stakeholder priorities

## Implications

An intentional focus on building capacity among interdisciplinary PHC trainees within the ACHRU research program created a culture that values interdisciplinary collaboration and understands the reciprocal relationship of investing in trainees. Capacity building efforts resulted in multiple impacts, included facilitating collaboration between established researchers, knowledge users, patients/caregivers, and trainees on actual projects; providing formal and informal mentorship opportunities for both research skill and career development; and creating experiential opportunities to learn about partnerships with diverse research stakeholders. An understanding of the importance of resources (including technology and support staff) and finances to support student fellowships (eg, capacity building integrated into grants) was also instrumental in facilitating ACHRU’s capacity building approach.

As a result of capacity building and mentorship within ACHRU, trainees have collectively coauthored 18 peer-reviewed publications and 3 government reports, with 15 additional peer-reviewed papers published or submitted with trainees as first authors. Trainees have also contributed to the development of numerous policy briefs and communication strategies targeting the public (eg, infographics, newsletters). Trainees have led or coauthored over 150 presentations at local, provincial, national, and international conferences. Trainees also had opportunities to actively engage in local community-based meetings with PHC practitioners, administrators, and members of the public. For example, trainees actively collaborated with Patient and Caregiver Research Partners to identify patient- and caregiver-relevant health priorities, gaps, and outcome measures for a transitional care intervention study, gaining valuable insight into the needs of older adults and their family/friend caregivers.

### Exemplar of trainee pathway

One doctoral trainee embedded her thesis in a multisite pragmatic randomized controlled trial. She developed a new evidence- and theory-based interprofessional education program as part of a complex intervention designed to support community-dwelling older adults poststroke. This trainee worked collaboratively with the interprofessional intervention teams to codesign, test, and implement coordinated care delivery. Embedding her work in the larger trial provided opportunities to engage in the research team and monthly team meetings with interventionists and their managers, to administer tools, and share results back with the teams to enhance team functioning. This trainee had opportunities to lead and coauthor publications, contribute to ministry reports, present her research, and colead the capacity building initiative during another trainee’s maternity leave.

## Limitations

Several implementation challenges were identified such as competing time commitments, limited office space, competing priorities, or variable levels of engagement by trainees. Subsequent initiatives would benefit from greater focus on formal evaluation including outcomes and impacts on future careers.

## Conclusion

Many ACHRU trainees have now completed their training programs; some are pursuing additional academic training, several have initiated careers in academia with early grant success, while others contribute to the health care system through leadership roles in policy and practice. ACHRU’s scientific leads, in turn, experienced reciprocity and a return on investment from capacity development efforts through mutual learning, enhanced academic outputs, and expertise brought by interdisciplinary PHC trainees.
